# Clinical outcome of postoperative highly conformal versus 3D conformal radiotherapy in patients with malignant pleural mesothelioma

**DOI:** 10.1186/1748-717X-9-32

**Published:** 2014-01-24

**Authors:** Jérôme Krayenbuehl, Peter Dimmerling, I Frank Ciernik, Oliver Riesterer

**Affiliations:** 1 Department of Radiation Oncology, Zurich University Hospital, Rämistrasse 100, 8091 Zurich, Switzerland; 2 Center for Clinical Research, University of Zurich, Zurich, Switzerland; 3 Department of Radiotherapy and Radiation Oncology, Dessau Municipal Hospital, Dessau, Germany

**Keywords:** Mesothelioma, Radiation therapy, Extrapleural pneumonectomy, Volumetric modulated arc therapy, Intensity modulated radiotherapy, Multimodal therapy

## Abstract

**Background:**

Radiotherapy (RT) is currently under investigation as part of a trimodality treatment of malignant pleural mesothelioma (MPM). The introduction of highly conformal radiotherapy (HCRT) technique improved dose delivery and target coverage in comparison to 3-dimensional conformal radiotherapy (3DCRT). The following study was undertaken to investigate the clinical outcome of both radiation techniques.

**Methods:**

Thirty-nine MPM patients were treated with neoadjuvant chemotherapy, extrapleural pneumonectomy (EPP) and adjuvant RT. Twenty-five patients were treated with 3DCRT, and 14 with HCRT (Intensity modulated radiotherapy or volumetric modulated arc therapy). Overall survival, disease free survival, locoregional recurrence and pattern of recurrence were assessed. A matched pair analysis was performed including 11 patients of each group.

**Results:**

After matching for gender, age, histology, tumor stage and resection status, HCRT seemed superior to 3DCRT with a local relapse rate of 27.3% compared to 72.7% after 3DCRT (*p* = 0.06). The median time to local relapse was increased by 49% with HCRT in comparison to 3DCRT from 10.9 ± 5.4 months to 16.2 ± 3.1 months (*p* = 0.06). The median overall survival was 22.3 ± 15.3 months for HCRT and 21.2 ± 9.2 months for 3DCRT (*p* = 0.57). Recurrence analysis showed that in-field local relapses occurred in previously underdosed regions of the tumor bed in 16% of patients treated with 3DCRT and in 0% of HCRT patients.

**Conclusions:**

The use of HCRT increases the probability of local control as compared to 3DCRT by improving target volume coverage. HCRT did not improve overall survival in this patient series due to the high rate of distant recurrences.

## Introduction

Malignant pleural mesothelioma (MPM) is a rare and aggressive malignancy associated with poor prognosis. Although MPM is often initially confined to the hemithorax, it has a high potential for metastatic spread in the course of disease [1]. The mainstay of treatment is surgery consisting of either pleurectomy/decortication (PD) or radical extrapleural pneumonectomy (EPP) in combination with cisplatin/pemetrexed and, in selected cases, postoperative radiotherapy [2-5]. The rationale to apply postoperative radiotherapy after EPP has been the high rate of local recurrence after EPP alone of about 40% [6].

The pattern of pleural dissemination, infiltrative growth and the manipulations within the chest cavity during surgery place the entire ipsilateral chest wall at high risk for post-surgical relapse, especially at the diaphragm insertion, the pericardium, mediastinum and bronchial stump. Technically, hemithoracic radiotherapy is challenging due to various reasons. Firstly, the size of the volume to be treated is large, and may cover up to six liters. Secondly, the target lies in close proximity to various organs at risk (OAR) such as the heart, ipsilateral kidney, liver, remaining lung, esophagus and/or spinal cord. Thirdly, the thoracic cavity has a complex shape with its costodiaphragmatic recess extending around the liver and the kidney. Previous publications showed that highly conformal radiotherapy (HCRT) such as intensity modulated radiotherapy (IMRT) or volumetric modulated arc therapy (VMAT) can improve the dose distribution in respect to target coverage and dose to OAR [7,8]. However to our knowledge there is no clinical study published that investigated and compared clinical outcome after both radiation techniques. In order to verify if the technical improvements introduced with IMRT or VMAT have translated into a clinical benefit, we evaluated the clinical outcome of MPM patients treated with chemotherapy, surgery and 3DCRT or HCRT at our institution.

## Material and methods

We reviewed the clinical outcome of 39 consecutive patients treated either with 3DCRT (25 patients) or HCRT (11 IMRT patients and 3 VMAT patients). Patient staging was established using FDG-PET/CT and/or conventional thoraco-abdominal CT. The patients with clinical stage T1-T3, N0-2, M0, R0-2 were treated with 3 cycles of preoperative chemotherapy (pemetrexed and cisplatin) followed by EPP and RT [7]. All histological subtypes were accepted for RT. Patients were not selected for this review if they had metastatic disease or a local relapse before the start of RT. The study was approved by the local ethics committee of the University Hospital of Zurich.

### Radiation techniques

The 3DCRT group was treated between 1999 and 2005. These patients were treated with 25 × 1.8 Gy = 45 Gy to the hemithorax and subsequently, in a second series, a boost of 7 × 1.8 Gy = 12.6 Gy was given to the incompletely resected area (total dose 57.6 Gy). Dose calculation was performed on Pinnacle planning system (Philips Medical Systems) for a linear accelerator (Clinac 2100C, Varian Medical Systems). Details of the treatment technique have previously been published [7].

HCRT has been used at our institution since 2005 for the treatment of MPM patients. Of the 14 patients treated with HCRT, 11 were treated with conventional static field IMRT and 3 patients with rotational IMRT (volumetric arc radiotherapy, i.e. Rapid Arc® in the present series).

IMRT and VMAT plans achieved similar dose distributions [9,10]. In the case of HCRT only one series was applied with 26 × 1.75 Gy = 45.5 Gy delivered to the hemithorax with a simultaneous integrated boost of 26 × 2.15 Gy = 55.9 Gy delivered to the R1/R2 region. Planning and dose calculation was performed on the Eclipse planning system (Varian Medical Systems, Palo Alto, CA) for a linear accelerator (Clinac 6EX or Trilogy, Varian Medical Systems). The treatment technique and dose-volume constraints have been previously published [7,10,11].

### Follow-Up and recurrence analysis

Patients were followed up every three to four months with clinical examinations and CT or PET/CT scans. Local tumor progression or recurrence was defined as an increasing radiographic abnormality within or partially within the irradiation field. Recurrence adjacent to the field border but not in-field was defined as marginal miss recurrence. Regional recurrence was defined as recurrence in close proximity but not within the irradiated field. Tumor recurrence in the contralateral hemithorax or abdominal cavity was classified as a distant recurrence [12]. All in-field recurrences were carefully analyzed by 2 of the authors (JK, PD), in order to assess if they occurred in previously underdosed areas by comparing the respective diagnostic image with the radiation therapy treatment plan.

### Statistics

All survival endpoints as well as tumor recurrence were measured from the date of treatment start (neoadjuvant chemotherapy) and were evaluated using the Kaplan Meier Method. In a subset of the cohort, a matched pair analysis was performed in order to compare outcome after 3DCRT and HCRT. For this analysis, the patients were matched for age, preoperative TNM, R and histology, and sex (except one pair).

## Results

Between 1999 and 2011, 39 patients were treated with neoadjuvant chemotherapy and EPP followed by RT. All follow up patients were deceased at the time of this study.

### Matched pair analysis

In the matched pair analysis, 11 HCRT and 11 3DCRT patients were matched based on tumor staging, resection status, tumor histology, age and gender (except one pair were the gender was not matched). In each group 3 patients had a tumor stage T1N0M0 with resection R0 and 8 patients, tumor stage T2N0M0 with resection R1. Tumor histology was epithelioid for 6 patients and biphasic for 5 patients in each group. The mean age was 59.6 years and 59.8 years for patient’s in the HCRT and 3DCRT group.

**Figure 1:**
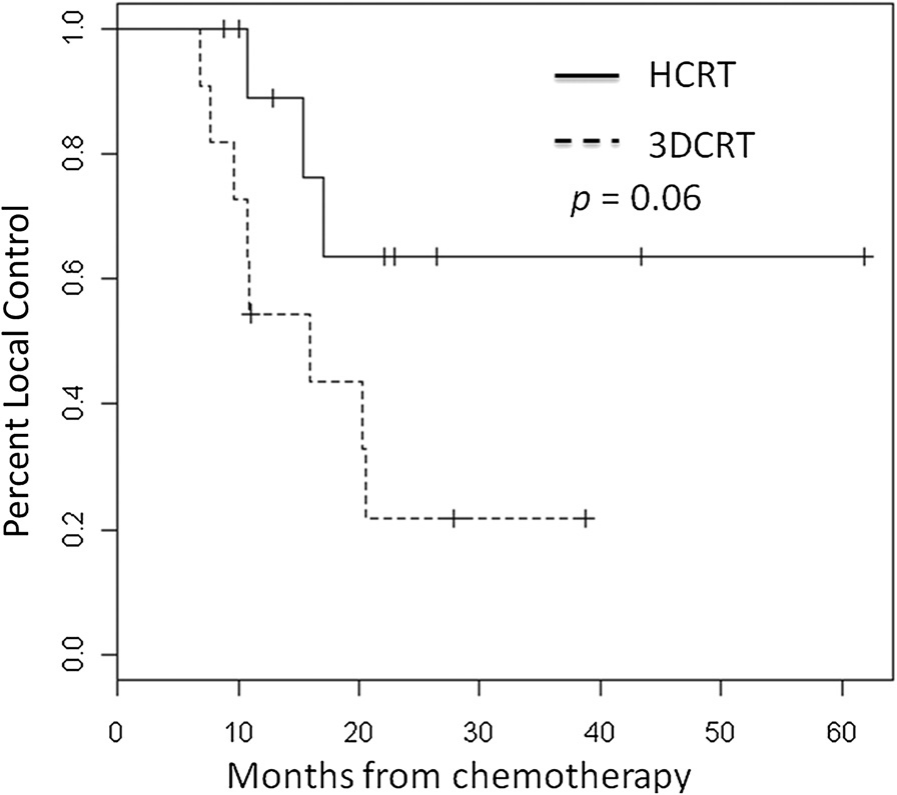
Local control for 11 matched modulated radiotherapy (HCRT) patients and 11 3-dimensional conformal radiotherapy (3DCRT) patients.

**Figure 2:**
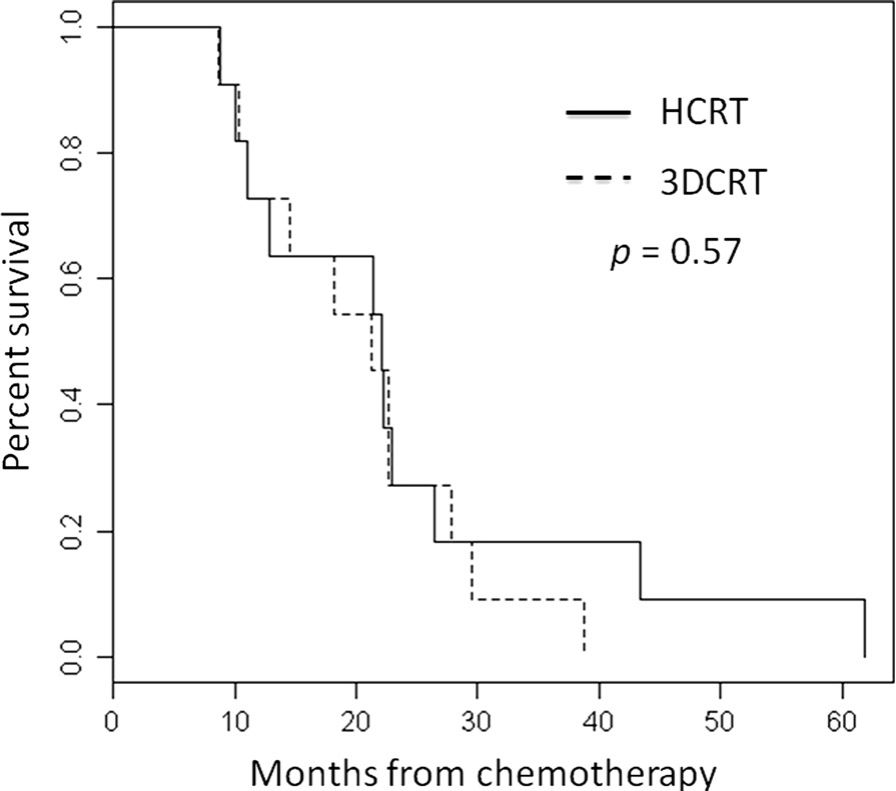
**Overall survival for 11 matched modulated radiotherapy (HCRT) patients and 11 3-dimensional conformal radiotherapy (3DCRT) patients**.

The median time to local relapse was increased by 49% with HCRT in comparison to 3DCRT from 10.9 ± 5.4 months to 16.2 ± 3.1 months (*p* = 0.06) as displayed in Figure 1. Three (27.3%) and eight patients (72.7%) had a local relapse after HCRT and 3DCRT respectively. Nine HCRT (81.8%) and nine 3DCRT (81.8%) patients developed metastases within a median time of 18.4 ± 10.7 months and 10.9 ± 8.6 months (*p* = 0.21). The difference in disease free survival between HCRT and 3DCRT was not significant (*p* = 0.72). The median overall survivals were 22.3 ± 15.3 months for HCRT and 21.2 ± 9.2 months for 3DCRT and are displayed in Figure 2 (*p* = 0.57).

### Outcome of the entire cohort

Fourteen HCRT and 25 3DCRT patients were treated and reviewed. Patient’s sex, age, tumor characteristics and resection are displayed on Table 1.

**Table 1:** Patient demographics and tumor characteristics of 39 patients who underwent neoadjuvant chemotherapy, extrapleural pneumonectomy and radiotherapy

**Characteristic**	**HCRT**	**3D-CRT**
	**n = 14**	**n = 25**
Sex		
• Male	13	22
• Female	1	3
Mean age (years)	61	61
Side		
• Right	9	15
• Left	5	10
Tumor histology		
• Epithelioid	8	17
• Biphasic	6	8
Initial Tumor stage		
• T1	3	15
• T2	10	10
• T3	1	0
Initial Nodal stage		
• N0	14	22
• N1	0	1
• N2	0	2
Resection		
• R0	4	8
• R1	9	15
• R2	1	2

*Abbreviation*: HCRT: Highly conformal modulated radiotherapy, 3DCRT: 3-dimensional conformal radiotherapy.

The median overall survival was 20.8 ± 14.4 months for the HCRT group, and 26.9 ± 11.8 months for the 3DCRT group (*p* = 0.48). In the HCRT group, 10 patients (71%) died of progressive disease and 4 patients (29%) due to intercurrent disease: one patient died of septic shock, one of acute myocardial infarction, one of progressive biventricular heart failure and another patient, who was well and without evidence of disease at two days before his sudden death most likely also died due to a cardiac event. In the 3DCRT group 24 patients (96%) died of progressive disease and one of septic shock (4%).

The local control rates were improved after HCRT (*p* = 0.30). Four HCRT patients (28.6%) suffered from locoregional relapse in comparison to 15 patients (60%) treated with 3DCRT.

### Analysis of tumor recurrence

For patients treated with HCRT, local relapse occurred in-field in 3 patients (21.4%), all within areas that had been treated with doses between 43 Gy to 59 Gy (according to our treatment planning protocol 95% of the prescribed 45 Gy (=43 Gy) should enclose the target volume, which in this case is the tumor bed of the hemithorax, Table 2) and none in a clearly underdosed region. One patient (7.1%) had a marginal miss recurrence at the field border (13 Gy). In the 3DCRT group, twelve patients (48%) had in-field recurrences in regions treated with doses between 30 Gy and 56 Gy (Table 3). Notably, in 16% of patients treated with 3DCRT (4/25) in-field recurrences occurred in regions that were covered with doses of only 30 to 43 Gy, instead of the prescribed ≥45 Gy. One patient (4.0%) had a marginal miss recurrence (18 Gy). In one patient with a regional recurrence (4.0%) the delivered dose was not possible to define because no diagnostic CT was available. In one patient (4.0%) the site of recurrence could not be determined because of missing radiographs during follow-up.

**Table 2:** Locoregional recurrences in patients who underwent highly conformal modulated radiotherapy

**Localization**	**Age/sex**	**Histology**	**Time to recurrence**	**Dose in recurrence region**
			**(Months)**	**(Gray)**
In-field recurrence	67/m	Epithelioid	10	54
In-field recurrence	62/m	Biphasic	15	56–59
In-field recurrence	64/m	Epithelioid	10	43–50
Marginal miss	65/m	Biphasic	17	13
Recurrence				

**Table 3:** Locoregional recurrences in patients who underwent 3D-conformal radiotherapy

**Localization**	**Age/sex**	**Histology**	**Time to recurrence**	**Dose in recurrence region**
			**(Months)**	**(Gray)**
In-field recurrence	61/m	Epithelioid	21	50
In-field recurrence	68/m	Biphasic	16	36
In-field recurrence	68/m	Epithelioid	13	30
In-field recurrence	65/m	Biphasic	19	56
In-field recurrence	65/m	Biphasic	16	50
In-field recurrence	50/m	Epithelioid	29	48
In-field recurrence	55/m	Biphasic	8	50
In-field recurrence	66/m	Biphasic	20	36, 50^1^
In-field recurrence	68/m	Epithelioid	11	32–43, 46–50^1^
In-field recurrence	58/m	Epithelioid	9	50
In-field recurrence	58/m	Epithelioid	11	50
In-field recurrence	65/m	Biphasic	19	30
Marginal miss recurrence	55/m	Epithelioid	19	18
Regional recurrence	62/m	Epithelioid	13	Not available
Unknown	56/m	Epithelioid	13	Not available

^1^The recurrence occurred in two separate regions.

Distant recurrences occurred in ten patients (71.4%) treated with HCRT and in twenty 3DCRT patients (80%). The median time to distant metastases was 18.4 ± 10.7 months in HCRT group and 16.7 ± 7.7 months in 3DCRT group (*p* = 0.7). In the HCRT group, distant metastases involved only the contralateral chest in three patients (30%) and only the abdominal cavity in three patients (30%). Both sites were affected by distant metastases in four further patients (40%).

## Discussion

We demonstrate in a retrospective analysis of patients with MPM and treated at our institution with trimodality therapy that the use of postoperative highly conformal radiation techniques (HCRT) reduces local recurrence in comparison to 3DCRT. A recurrence analysis showed that in the case of 3DCRT 4 of 25 patients (16%) had a local recurrence in regions that were clearly underdosed according to current radiation protocols (doses ≥ 45 Gy are recommended, e.g. SAKK 17/04) in contrast to 0% of patients treated with HCRT. This supports the hypothesis that HCRT should improve local control in comparison to 3DCRT by improving target volume coverage. In our study patients treated with HCRT showed a tendency for improved progression free survival and local relapse free survival but did not benefit in terms of overall survival due to the high rates of distant relapses.

Local control is important in patients with MPM for symptom control, but also because some patients might benefit in terms of improved overall survival. Better local control after HCRT did not translate into improved overall survival in our patient series. Remarkably, the rate of death due to intercurrent disease, most often cardiac events, was higher after HCRT (29%) in comparison to 3DCRT (4%). Since cardiac sparing is rather improved with HCRT the most likely explanation for this difference is patient selection. The urgent research question, if postoperative radiotherapy impacts on overall survival after EPP, is addressed by a randomized study currently conducted in Switzerland, SAKK 1704. Patient accrual for this study was terminated in 2012 and the results are awaited.

Even after trimodality treatment local recurrence remains high in some patient series. In a retrospective series of 49 patients treated with 3D-conformal RT after EPP and chemotherapy 67% of all recurrences included the ipsilateral hemithorax and 25% of all recurrences were local only [12]. Therefore improvement of radiotherapy is mandatory. In recent years radiotherapy has made enormous technical advances. More sophisticated highly conformal radiation techniques (HCRT) such as IMRT or rotational RT (VMAT) have become available and substituted for the older 3DCRT technique. The use of HCRT enables improvement in the dose distribution and target volume coverage. This is because with HCRT even complex target volumes such as the tumor bed of the costodiaphragmatic recess or the pericardium can be treated without or with little dose compromise and at the same time with optimal sparing of the normal tissue due to a steeper dose fall-off. Thus, the use of HCRT should intuitively improve treatment outcome in terms of local tumor control. Our data suggest indeed, that the use of HCRT bears considerable potential to improve on hemi-thoracic tumor control rates most likely due to improved target volume coverage.

The poor local control rates and high rates of in-field recurrences following 3DCRT in our cohort may be due to suboptimal dose coverage or the restriction of the target volume to avoid critical organs, both limitations inherent to the technique. After 3DCRT 4/24 (16.6%) in-field recurrences occurred in regions covered with only 30–43 Gy. In the case of 3DCRT mixed beams of photons and electrons were used to optimize dose coverage. The match of these beams often causes cold and hot spots of dose coverage. Poor matching during daily treatment can result in >20% dose inhomogeneity in the junction area [7]. In addition, as the spinal cord is blocked when the tolerance dose of 45 Gy is reached, insufficient dose delivery to parts of the mediastinum has been observed, resulting in underdosage to the tumor bed [7].

Favorable tumor control after IMRT as part of a trimodality therapy has previously been reported by Rice *et al*. [13]. The median overall survival of their 61 patients treated was 14.2 months with a locoregional recurrence rate of 13% and only 5% local in-field recurrences reported. The median dose prescribed was only 45 Gy, and half of all patients received doses even less than 45 Gy. The reason for the comparatively higher local control rate reported by Rice et al. in comparison to our study remains unclear. It may be explained by patient selection and the comparatively short median overall survival of 14.2 months in comparison to 20.8 months in the present series and by the retrospective study design. The shorter median overall survival reported by Rice et al. could be caused by more advanced tumor stages (40 T3, 8 T4, 26 N2), more aggressive subtypes (14 biphasic, 4 sarcomatoid) and the fact that neoadjuvant chemotherapy was not routinely administered.

With regard to toxicity the major dose limiting organ for postoperative radiotherapy of MPM is the contralateral lung. Lung complications such as radiation pneumonitis are likely to be higher with multi-field techniques such as IMRT or VMAT in comparison to 3DCRT, where opposed beams from 0 and 180 degrees are usually used, thereby optimally sparing the contralateral lung. With regard to dose escalation and lung sparing surgery, protons might prove superior to IMRT/VMAT. Severe complications of the lung with grade 4 and 5 pneumonitis after IMRT have been reported [7,12]. Since then, special attention to the contralateral lung dose has been given during the treatment planning process and pneumonitis rates should be lower today. Intuitively, the use of HCRT should reduce toxicity and complication probabilities of esophagus, heart, liver and kidney, however no data with regard to these toxicity endpoints comparing both treatment techniques are available.

In recent years, the need for extensive surgery has been questioned, and less radical surgery has been advocated such as pleurectomy/decortication. In the context of reduced surgery, the anatomical situation makes it difficult for RT to be applied to the entire pleural space, however, it can still be considered as a targeted local postoperative option in case of incomplete resection. Future clinical studies are required to define the role of radiotherapy in combination with lung sparing surgery.

## Conclusions

In summary, the use of HCRT for treatment of patients with MPM after EPP is likely to improve local control rates. The local control improvement did not improve the overall survival due to the high rates of distant relapses in this series. Further improvement of trimodal or systemic therapy is required to tackle the high risk of distant recurrences.

## Competing interests

The authors declare that they have no competing interests.

## Authors’ contributions

JK, PD and OR were responsible for the study design and implementation. JK and PD performed the data analysis. JK, PD, IC and OR contributed to the implementation and manuscript writing. All authors read and approved the final manuscript.
